# Diversity of the Seedborne Fungi and Pathogenicity of *Fusarium* Species Associated with Intercropped Soybean

**DOI:** 10.3390/pathogens9070531

**Published:** 2020-07-01

**Authors:** Xiaoli Chang, Hongju Li, Muhammd Naeem, Xiaoling Wu, Taiwen Yong, Chun Song, Taiguo Liu, Wanquan Chen, Wenyu Yang

**Affiliations:** 1 College of Agronomy & Sichuan Engineering Research Center for Crop Strip Intercropping system, Sichuan Agricultural University, Chengdu 611130, China; xl_chang14042@sicau.edu.cn (X.C.); lihongju@stu.sicau.edu.cn (H.L.); muhammdnaeem201@gmail.com (M.N.); wulx@sicau.edu.cn (X.W.); yongtaiwen@sicau.edu.cn (T.Y.); songchun@sicau.edu.cn (C.S.); 2 State Key Laboratory for Biology of Plant Diseases and Insect Pests, Institute of Plant Protection, Chinese Academy of Agricultural Sciences, Beijing 100193, China; liutaiguo@caas.cn; 3 National Agricultural Experimental Station for Plant Protection, Ministry of Agriculture and Rural Affairs, Gansu 741200, China

**Keywords:** soybean, seedborne fungi, *Fusarium* species, isolation frequency, pathogenicity

## Abstract

Maize/soybean relay strip intercropping has been widely practiced in Southwest China due to its high productivity and effective application of agricultural resources; however, several seedborne diseases such as seedling blight, pod and seed decay are frequently observed causing severe yield loss and low seed quality. So far, the population and pathogenicity of the seedborne fungi associated with intercropped soybean remain unexplored. In this study, seeds of 12 soybean cultivars screened for intercropping were collected from three growing regions in Sichuan Province of Southwest China, and the seedborne fungi were isolated from the surface-sterilized seeds. Based on sequence analysis of ribosomal DNA internal transcribed spacer (*rDNA ITS*)*,* 148 isolates were identified into 13 fungal genera, among which *Fusarium* covered 55.0% as the biggest population followed by *Colletotrichum*. Furthermore, *Fusarium* isolates were classified into five distinct species comprising *F. fujikuroi*, *F. proliferatum*, *F. verticillioides*, *F. asiaticum* and *F. incarnatum* through sequence analysis of translation elongation factor 1 alpha (*EF-1α*) and DNA-directed RNA ploymerase II second largest subunit (*RPB2*). Among them, *F. fujikuroi* accounted for 51.22% (42/82) and was isolated from 91.7% (11/12) soybean varieties. Pathogenicity assay showed that five *Fusarium* species were able to infect the seeds of soybean cultivar “Nandou12” and caused water-soaked or rot symptoms, while *F. fujikuroi* and *F. asiaticum* had much higher aggressiveness than other species with significant reductions of seed fresh weight and germination percentage. Accordingly, this study indicates that *Fusarium* species are the dominant seedborne fungi in the intercropped soybean in Sichuan, China, and this provides some useful references for the effective management of seedborne fungal diseases as well as soybean resistance breeding in maize/soybean relay strip intercropping.

## 1. Introduction

Soybean (*Glycine max* L.) is one of the most important oil seeds and economic crops across the world. In China, the annual demand for the domestic soybean has been rising, up to 110 million metric tons, accounting for 31.18% of global consumption, but more than 90% consumption is still dependent on the overseas import because of limited domestic production that was averaged about 16 million metric tons in 2018–2019 (USDA, https://www.usda.gov/oce/commodity/wasde/). Several seedborne fungal diseases have been known as one major limiting factor of soybean production [1,2,3,4]. Most of these seedborne fungi are facultatively parasitic in soybean, and they can infect and colonize seeds, which often cause seedling blight, pod and seed decay; significantly affect the yield; and reduce the germination, vigor and quality of seeds [2,5,6]. On the other hand, infected seeds may serve as a source of local or long-distance dispersal of pathogens, thereby providing the potential for the spread of seedborne diseases [5,7]. Although the saprophytic seedborne fungi normally have no direct damage to soybean seeds, they can produce mycotoxins in stored seeds and bring a health risk to humans and livestock [8,9,10]. Currently, management of seedborne diseases has been conducted using a combination of fungicides, intercropping or rotation with non-host crops, early harvest and application of resistant cultivars to minimize the yield loss [11,12,13,14,15,16]. 

Regarding to the seedborne fungi, the species in the genera of *Colletotrichum*, *Phomopsis*, *Fusarium* and *Alternaria* are the most frequently isolated fungi from soybean seeds [1,17,18,19,20,21]. Among them, *Phomopsis longicolla* (syn. *Diaporthe longicolla*) is the primary causal agent of *Phomopsis* seed decay (PSD) in soybean and has become one of the most economically important seed diseases in soybean [15,18]. Many other species such as *D. phaseolorum* var. *sojae* and *Diaporthe phaseolorum* var. *caulivora* in *Diaporthe/Phomopsis* complex can also cause seed decay [1,19,22]. Besides *Phomopsis* spp., several *Fusarium* species have frequently been reported to deteriorate the seed quality, as well as cause root rot, seed decay and pod and seedling blight [10,23,24,25]. In North America, at least 14 species in the *Fusarium* genus have reported in soybean seeds [2,17]. Among them, the infection of *F. verticillioides* changed flavonoids content in seeds and resulted in poor seed quality [3,25]. In China, *Fusarium* species have the potential to cause seed and pod decay and root rot in soybean [4,26,27]. Although the importance of *Fusarium* species to soybean production has been documented, the pathogenicity of soybean seedborne *Fusarium* species is not fully understood.

Many studies have demonstrated that delayed harvest coupled with unusually warm and moist weather conditions are favorable for pod and seed invasion [3,28,29]. Recently, a maize/soybean relay intercropping system is widely adopted in the southwestern areas of China for dietary fibers and food [30], and it has gradually been accepted because of its obvious advantages on efficient use of resources, improving soil quality and crop production and suppressing the field weeds [31,32]. However, the climate is often characterized by the continuous rainfall and high humidity at the pre- and post-harvest stage of soybean, which causes severe seed decay and affects the yield and quality of soybean. Until now, there is no report on the population of seedborne fungi in intercropped soybean in Southwest China that limits the management of soybean seedborne diseases. 

The objectives of this work were to identify the population of the seedborne fungi isolated from different soybean varieties used in maize/soybean relay strip intercropping in Southwest China and to evaluate the pathogenicity and isolation frequency of *Fusarium* species and their impacts on seed germination. Hence, this work will be critical for the effective management of soybean seedborne diseases and soybean resistance breeding.

## 2. Results

### 2.1. Population Identification of the Seedborne Fungi Associated with Intercropped Soybean

**Figure 1:**
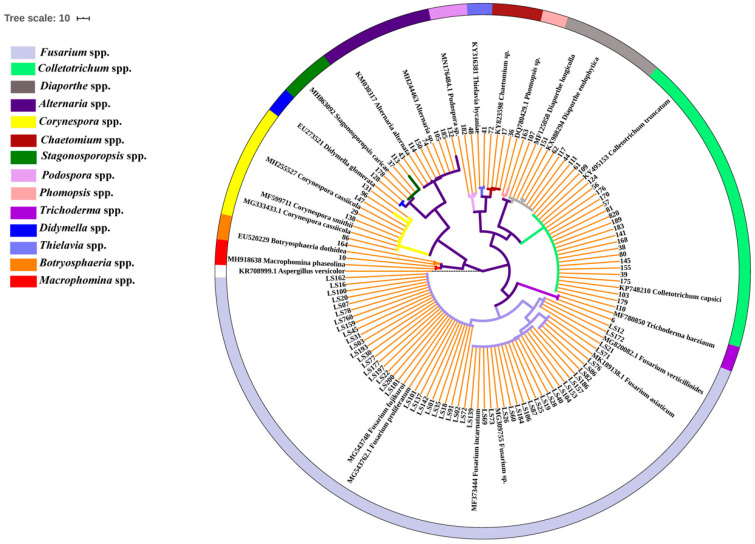
Phylogenetic tree of the seedborne fungi isolated from soybean based on *rDNA ITS* gene. The phylogenetic tree was constructed using a maximum-parsimony method by MEGA 7.0.26. Bootstrap support values were from 1000 replications. Tree branches with bootstrap value >70 are shown in the phylogenetic tree. The *rDNA ITS* sequences of referred isolates were obtained from GenBank, and *Aspergillus versicolor* (KR708999.1) was selected as an outgroup. Fungal genera were marked with different colors and indicated in the left color bars.

**Figure 2:**
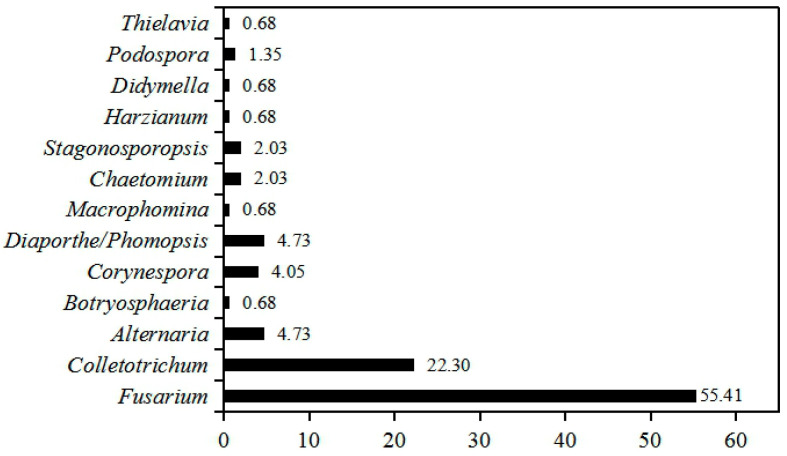
Isolation frequency of different seedborne fungal genera associated with soybean seeds. The isolation frequency was calculated using the percentage of isolates numbers each genus in the total fungal isolates obtained. The number in different bars indicate the isolation frequency each genus.

To uncover the seedborne fungal population associated with intercropped soybean, 148 isolates were recovered from the surface-sterilized seeds of 12 soybean cultivars. The partial sequences of ribosomal DNA internal transcribed spacer (*rDNA ITS)* of these isolates were amplified and analyzed using BLAST in NCBI database, and the results show that they had 99–100% identities with *Fusarium* spp., *Colletotrichum* spp., *Alternaria* spp., *Corynespora* spp., *Diaporthe/Phomopsis* spp., *Stagonosporopsis* spp., *Chaetomium* spp., *Podospora* spp., *Botryosphaeria* spp., *Thielavia* spp., *Macrophomina* spp., *Harzianum* spp. and *Didymella* spp. (Table S1). Phylogenetic analysis showed these isolates were clearly classified into different clades in the maximum-parsimony tree (Figure 1). Among them, isolates of *Fusarium* genus accounted for 55.00% (82/148) of all fungal isolates, followed by *Colletotrichum* for 22.30% (33/148), while *Alternaria* and *Diaporthe/Phomopsis* displayed the same isolation percentage (4.73%, 7/148) (Figure 2). In addition, the isolation frequency of other genera was less than 4.05%. Hence, *Fusarium* was the biggest seedborne fungal genus associated with intercropped soybean, and *Colletotrichum* was the second one. 

### 2.2. Identification of Fusarium Species Associated with Soybean Seeds Based on EF-1α and RPB2 Genes

**Figure 3:**
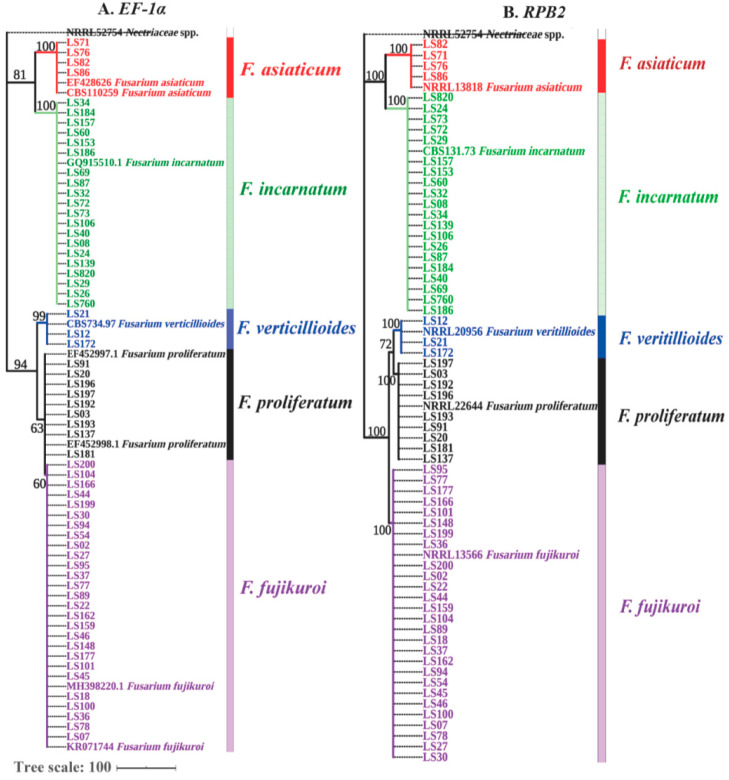
Phylogenetic trees constructed based on *EF-1α* and *RPB2* genes. In total, 73 representative *Fusarium* isolates, including 27 isolates of *F. fujikuroi*, 20 of *F. incarnatum*, 9 of *F. proliferatum*, 4 of *F. asiaticum* and 3 *F. verticillioides,* were used for both phylogenetic trees, while *Nectriaceae* sp. (NRRL52754) was used as the outgroup. those referred sequences and were obtained from *Fusarium MLST* and *Fusarium* ID databases. (**A**) The maximum-parsimony tree of *EF-1α* gene was constructed by MEGA 7.0.26, and the tree parameters were 138 steps for TL, 0.934 for CI, 0.996 for RI and 0.960 for RCI. (**B**) The maximum-parsimony tree of *RPB2* gene, and the tree parameters were 568 steps for TL, 0.897 for CI, 0.992 for RI and 0.943 for RCI. Bootstrap support values were obtained from 1000 replications. The bootstrap values >60 are shown in the trees. The tree scale was 100. Different colors indicate different *Fusarium* species.

As shown above, *Fusarium* was found as the predominant genus of soybean seedborne fungi. To further verify *Fusarium* species, 82 *Fusarium* isolates were identified through a combination analysis of *EF-1α* and *RPB2* genes. BLASTn analysis showed that these *Fusarium* isolates showed over 99% sequence similarity with *F. fujikuroi*, *F. proliferatum*, *F. verticillioides*, *F. incarnatum* and *F. asiaticum* on the databases of *Fusarium* ID and *Fusarium MLST* (Table S1). For phylogenetic analysis, a maximum-parsimony tree based on *EF-1α* gene was constructed including 73 out of 82 *Fusarium* isolates, 6 referred isolates and 1 outgroup isolate *Nectriaceae* sp. (NRRL52754). As shown in Figure 3A (TL = 138 steps, CI = 0.934, RI = 0.996 and RCI = 0.960), *F. verticillioides*, *F. incarnatum* and *F. asiaticum* were clearly classified into single clade, but *F. fujikuroi* and *F. proliferatum* had close genetic relationship and shared one big branch. To further discriminate *F. fujikuroi* and *F. proliferatum*, another maximum-parsimony tree based on *RPB2* gene was constructed, as shown in Figure 3B (TL = 568 steps, CI = 0.897, RI = 0.992 and RCI = 0.943), where all five *Fusarium* species were obviously branched into five separate clades. Thus, a combined sequence analysis of *EF-1α* and *RPB2* genes confirmed that *F. fujikuroi*, *F. proliferatum*, *F. verticillioides*, *F. incarnatum* and *F. asiaticum* were the seedborne *Fusarium* species associated with intercropped soybean.

### 2.3. Isolation Frequency of Fusarium Species and Its Diversity Correlated with Soybean Varieties

**Figure 4:**
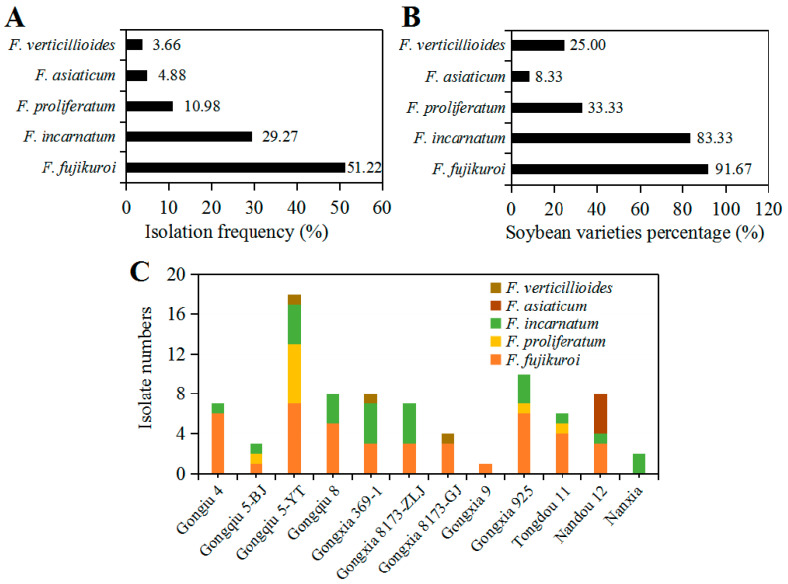
Isolation frequency and variety-specificity of *Fusarium* species associated with soybean seeds: (**A**) Tsolation percentage of each *Fusarium* species in total *Fusarium* isolates; (**B**) the percentage of soybean varieties colonized by *Fusarium* species; and (**C**) isolate numbers of *Fusarium* species recovered from different soybean varieties. The difference of isolation percentage and soybean varieties percentage was analyzed by Fisher’s exact test.

As shown in Figure 4A, comparison of the isolation percentage of five *Fusarium* species showed *F. fujikuroi* covered up to 51.22% of all *Fusarium* isolates followed by 29.27% for *F. incarnatum* and 10.98% for *F. proliferatum*, while *F. asiaticum* and *F. verticillioides* accounted for 4.88% and 3.66%, respectively. Regarding to soybean varieties, 82 *Fusarium* isolates were obtained from 12 different soybean varieties. Among them, *F. fujikuroi* as the most dominant pathogen was isolated from 91.67% soybean varieties followed by *F. incarnatum* from 83.33% varieties, while *F. asiaticum* was able to infect only one variety of soybean, accounting for 8.33% of total soybean varieties (Figure 4B). Additionally, there was a distinct difference in the diversity of *Fusarium* species regarding soybean varieties (P = 0.0325, Fisher’s exact test). Gongxia9 and Nanxia were only colonized by *F. proliferatum* and *F. incarnatum*, respectively, and showed relatively high resistance to *Fusarium* species (Figure 4C). In contrast, four species of *Fusarium* genus were isolated from Gongqiu 5-YT, thus showing low resistance to *Fusarium* species (Figure 6C). In general, the diversity of *Fusarium* species was correlated with soybean varieties cultivated in Sichuan Province of Southwest China.

### 2.4. Pathogenicity of the Seedborne Fusarium Species Associated with Intercropped Soybean

**Figure 5:**
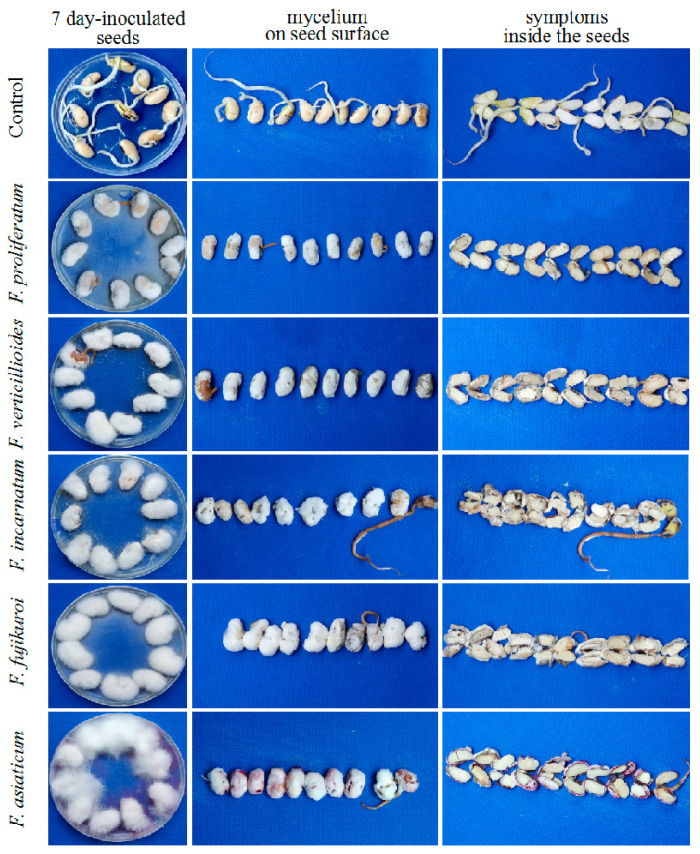
Symptoms infected by the representative isolates of *Fusarium* species associated with soybean seed decay. The representative isolates include *F. proliferatum* LS03, *F. fujikuroi* LS77, *F. verticillioides* LS12, *F. asiatcum* LS71 and *F. incarnatum* LS72. Control means the seeds inoculated with PDA plugs instead of *Fusarium* isolates. After seven-day culture on PDA plates, mycelium growth of *Fusarium* species on the seed surface and the infection symptoms inside the seeds were observed.

**Figure 6:**
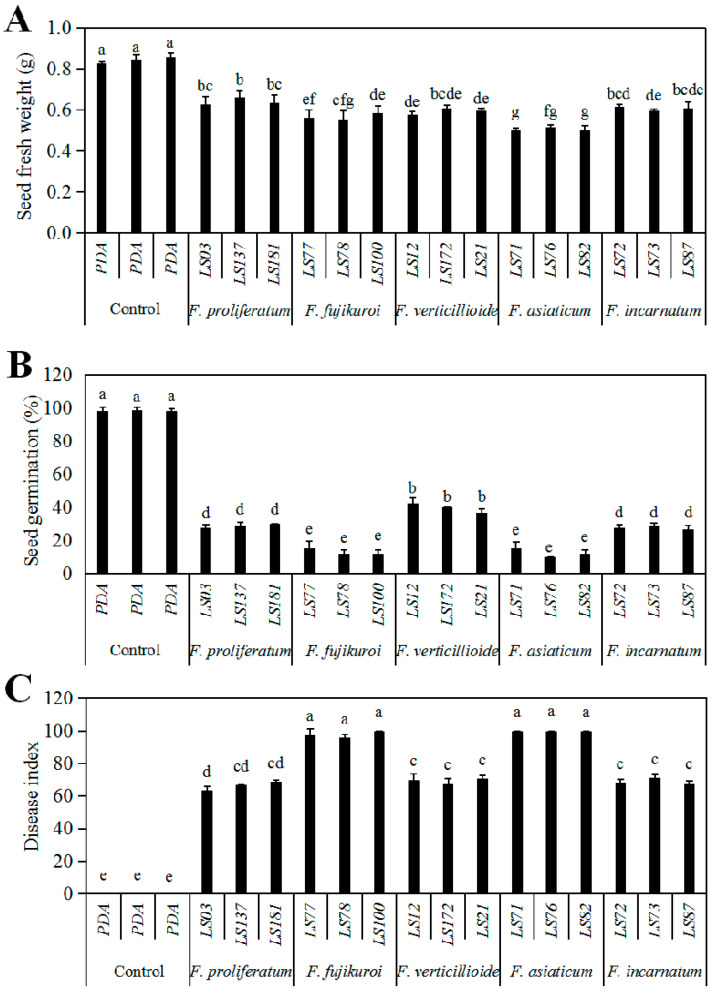
Growth and disease parameters of soybean cultivar Nandou12 after inoculated with the representative isolates of *Fusarium* species. The representative isolates include *F. proliferatum* LS03, LS137 and LS181; *F. fujikuroi* LS77, LS78 and LS100; *F. verticillioides* LS12, LS172 and LS21; *F. asiaticum* LS71, LS76 and LS82; and *F. incarnatum* LS72, LS73 and LS87. PDA was the control, meaning the seeds inoculated with PDA plugs instead of *Fusarium* isolates. Means of germination percentage (**A**) and fresh weight (**B**) of soybean seeds were tested after seven-day inoculation, and disease indices (**C**) were calculated according to disease grade by the statistical analysis using SPSS ANOVA. The data correlation was conducted by generalized linear model (GLM) with quasipoisson distribution for residuals, and statistical analysis was performed by Duncan’s test using SPSS Statistics 21. Different lowercase letters indicate the significant difference at the level of 0.01.

The pathogenicity of the seedborne *Fusarium* species of soybean were tested using spore suspension of the representative isolates on soybean cultivar “Nandou12”. After seven-day inoculation, the symptoms of white or pink fluffy aerial mycelia on seed surface, water-soaked and even brown decay inside the seeds were observed, while the control seeds showed no significant symptoms (Figure 5). Infection of all representative isolates significantly reduced fresh weight (Figure 6A) and germination percentage as of soybean seeds compared to the control; among them, *F. asiaticum* and *F. fujikuroi* caused the lower germination percentage (10.00–11.67% and 11.67−18.67%, respectively) than other species (Figure 6B). Additionally, both *F. asiaticum* and *F. fujikuroi* also caused the highest disease index (DI) (Figure 6C) by developing the severe water-soaked and decay symptoms inside the seeds (Figure 5). The DSI of *F. incarnatum* was 68.1 as the moderate aggressive species, whereas it was 63.33−68.72 for *F. proliferatum*. Thus, our results demonstrate that *F. asiaticum* and *F. fujikuroi* had the highest aggressiveness on soybean seeds, and these seedborne *Fusarium* species had some negative effects on seed fresh weight and seed germination. 

## 3. Discussion

The seedborne fungi may not only decrease seed quality but also provide the primary inoculum for diverse soybean diseases in soybean [2,3]. Meanwhile, the movement of these fungi through infected seeds brings an important challenge to the global soybean production as seeds may travel across the world [2,33]. Identification of the seedborne fungi associated with soybean provides the first step toward significant improvements in the management of seedborne diseases and the breeding of resistant cultivar. 

In this study, we identified 13 genera of the seedborne fungi and found *Fusarium* as the highest isolated genus (55.00%, 82/148), which was followed by *Colletotrichum*, *Alternaria*, *Diaporthe/Phomopsis* and nine other genera with less isolation frequency. Except for *Colletotrichum*, our results are nearly consistent with previous studies [34,35], but Escamilla et al. [34] identified seven species in six fungal genera from the commercial sprout soybean seeds in the United States and among them the most frequent genera were *Alternaria*, *Diaphorte* and *Fusarium*. However, Wei et al. [36] found that the genus *Penicillium* was the dominant population of seedborne fungi in different soybean cultivars in several soybean-producing regions of China, which were followed by *Fusarium* and *Aspergillus.* This difference might be associated with special maize/soybean relay strip intercropping pattern in Sichuan province of Southwest China. These genera have previously been reported as the important pathogens of several soybean diseases, such as seedling blight, root rot and pod and seed decay by *Fusarium* spp. [4,10,25,27,37]; seedling blight and seed decay caused by *Diaporthe/Phomopsis* species complex [15,18]; soybean anthracnose caused by *Colletotrichum* spp. [21,38]; and leaf spot by *Alternaria* spp. [39]. Thus, the diversity of the seedborne fungal genera associated with intercropped soybean indicates a big risk for soybean diseases in Southwest China. 

In general, the frequency of seedborne pathogens among and within seed samples may vary depending on geographical location, host genotype and agricultural practices [2]. Previous studies showed that only 33% of soybean seeds were infected by *Fusarium* species in Kansas [37]. Among nine *Fusarium* species found in soybean seeds, *F. semitectum* was the most frequent species in Kansas with the isolation frequency of 56.42% followed by *F. proliferatum* (19.25%) and *F. verticillioides* (13.20%), while *F. equiseti* (2.80%), *F. fujikuroi* (1.95%) and *F. graminearum* (0.33%) were isolated in low frequency among naturally infected seeds [25,40,41]. In our study, five *Fusarium* species were identified from intercropped soybean seeds, but they had different isolation frequency. *Fusarium fujikuroi* was found as the most frequently encountered species (51.22%), followed by *F. incarnatum* and *F. proliferatum* (Figure 4A). The high frequency of these two species from soybean seeds may be correlated with local soybean planting pattern in Southwest China because *F. fujikuroi* and *F. proliferatum* were reported as the most common *Fusarium* species causing maize stalk and ear rot [42]. In Sichuan Province of Southwest China, the soybean producers predominantly use the maize relay strip intercropped with soybean, and in this pattern maize plants after harvest are often used to cover the fields for keeping soil moist and avoiding weeds growth [32], which in turn provides favorable conditions for the inoculum accumulation and cycle infection of the pathogenic *Fusarium* species on subsequent soybean. Similarly, *F. asiaticum* as the predominant pathogen of *Fusarium* head blight (FHB) in this region [43], was also recovered from soybean seeds in our study and it showed the same high pathogenicity as *F. fujikuroi*, suggesting that the host range of *F. asiaticum* has shifted onto soybean [11]. Furthermore, we also found that *Fusarium* species were able to be isolated from the seeds of all 12 soybean varieties, but different species had some certain host specificity. In particular, both Nanxia and Gongxia9 varieties were only colonized by single *Fusarium* species, indicating that a correlation between the specificity of *Fusarium* species and soybean varieties. 

Seeds infected with pathogenic fungi can decrease seed germination and vigor, resulting in reduced seed quality [5,41,44]. In our study, five species of *Fusarium* genus significantly reduced the germination percentage of artificially-inoculated soybean seeds when compared with control, and *F. asiaticum* and *F. fujikuroi* had obvious negative effects on soybean seeds. This is supported by previous study that soybean seedborne *F. fujikuroi* significantly reduced rice seed germination, promoted post-emergent damping off, and caused inter-node elongation [41]. *Fusarium asiaticum* and *F. proliferatum* which were reported to be highly toxic to grains [45,46], thus they had the potential to decrease soybean seed germination and vigor in this study. Pedrozo and Little [25] showed that the potential of *F. verticillioides* to decrease soybean seed quality was dependent upon the inoculum potential present in the seeds. In the current study, *F. incarnatum* was observed as the moderate pathogenic fungus and had negative effects on seed germination and fresh seed weight, which is also consistent with our previous study [4]. The different pathogenicity of *Fusarium* species might be explained by their specific virulence mechanism. As hemibiotrophic pathogens, *Fusarium* can produce several cell wall degrading enzymes, such as cellulases, pectinases and xlylanases, to penetrate into host cell, and they also can synthesize several mycotoxins including trichotecenes, fumonisins and zearalenone to hijack host secondary metabolic pathways and even cause plant cell death [47,48]. However, the phenotypical characterization of our isolated *Fusarium* spp. regarding to the production of different virulence factors are not analyzed and should be focused on in future work in order to explain the correlation of virulence factor production of *Fusarium* species, pathogenicity and soybean varieties. Moreover, considering that only one soybean cultivar, “Nandou12” was used for the pathogenicity test in this study, it is necessary to analyze the relationship of pathogenicity of *Fusarium* species and seed quality of other intercropped soybean cultivars. 

Additionally, as expected, this study is basically consistent with our previous research on the characterization of *Fusarium* species associated with intercropped soybean pod decay in which *F. fujikuroi*, *F. incarnatum*-equiseti complex species, *F. proliferatum* and *F. graminearum* were identified [4], thus a close relationship of *Fusarium* species infection exist between soybean pods and seeds. This was also supported by Liu et al. [3] who found that *F. verticillioides* was the dominant species of field pod mold in Sichuan, China. There is evidence suggesting that certain fungi initially invade pods and subsequently progress into developing seeds [17]. Thus, to uncover the infection mechanism, additional useful information could be obtained from the studies of pod and seed microbiome during their development and maturation. 

## 4. Materials and Methods

### 4.1. Samples Collection and Isolation of Seedborne Fungi

In 2018, seeds were collected at harvest time from 12 different soybean varieties used in the maize/soybean relay strip intercropping in Chongzhou (103°70′ E, 30°53′ N) and Zigong (104°20′ E, 29°38′ N) and Nanchong (106°11′ E, 30°84′ N) of Sichuan Province, China (Table S1). The symptomatic seeds characterized by rotten, discolored and obvious disease spots with chalky appearance were surface-disinfested in 1% sodium hypochlorite for 3 min, rinsed three times in sterile distilled water and cultured on potato dextrose agar (PDA, 200 g∙L^−1^ potato, 15 g∙L^−1^ agar and 10 g∙L^−1^ glucose anhydrous) containing 50 μg∙mL^−1^ streptomycin according to Li and Chen [14] with minor revisions. Assay cultures were incubated at 25 ± 2 °C in the darkness for 7−10 days until fungal mycelium grew on seed surface. All cultures were purified and established by single spore isolation [27]. 

### 4.2. PCR Amplification of rDNA ITS, EF-1α and RPB2 Sequences

All fungal isolates were cultured on PDA at 25 ± 2 °C in the dark for 7 days, and the aerial mycelia were scraped directly from the colonies. The genomic DNA of all isolates was extracted using SP fungal DNA extraction kit (Aidlab Biotech, China) according to the manufacturer’s protocols. Quantity and quality of total DNA were estimated using a Thermo Scientific NanoDrop™ 2000 Spectrophotometer (Delaware, USA). The partial sequences of the ribosomal internal transcribed spacer region (*rDNA ITS*) as an official fungi locus [49,50] was initially used to identify the seedborne fungi at the genus level. For the accurate identification of *Fusarium* species, partial sequences of translation elongation factor 1 alpha (*EF-1α*) and DNA-directed RNA polymerase II second largest subunit (*RPB2*) genes [50,51,52] were specifically amplified and analyzed. PCR reaction was conducted in a final volume of 50 μL containing total genomic DNA (2 μL), each primer (2 μL) (10 μM), Taq PCR Mastermix (Sangon Biotech, Shanghai, China) (25 μL) and DNase free water (19 μL). The *rDNA ITS* partial region was amplified using the primer pairs ITS1/ITS4 as reported White et al. [49], and amplification parameters were 5 min at 94 °C of initial denaturation, followed by denaturation of 35 cycles at 94 °C for 45 s, annealing at 58 °C for 45 s and initial extension at 72 °C for 1 min with a final extension of 10 min at 72 °C. Sequences of *EF-1α* and *RPB2* were amplified using the primer pairs EF1/EF2 [50] and RPB2-5f2/RPB2-7cr [53], respectively, and PCR amplification were conditioned by 5 min at 94 °C, followed by 35 cycles of denaturation at 94 °C for 30 s, annealing at 55 °C for 30 s, initial extension at 72 °C for 1 min and kept at 72 °C for 10 min. Amplification was carried out using S-1000TM Thermal Cycler (Bio-Rad, Foster City, California, USA), and PCR products were detected by a 1% (*w*/*v*) agarose gel. All sequences were analyzed by an ABI-PRISM3730 automatic sequencer (Applied Biosystems, Foster, USA) using the same primer pairs as PCR amplification. 

### 4.3. Phylogenetic Analysis

Sequence analysis of *rDNA ITS* was firstly performed using BLASTn on the NCBI database, while those sequences of *EF-1α* and *RPB2* for *Fusarium* species were compared to the databases of *Fusarium* ID (http://isolate.Fusariumdb.org/guide.php) and *Fusarium MLST* (http://www.wi.knaw.nl/Fusarium/Biolomics.aspx). Sequences with maximum nucleotide similarity were downloaded as referred sequences. All sequences of *Fusarium* isolates and referred sequences from *Fusarium* ID or *Fusarium MLST* were edited and aligned with Clustal X 1.83 (http://www.sgi.com/industries/sciences/chembio/resources/clustalw/parallel_clustalw.html), while characters were weighed equally. The maximum-parsimony phylogenetic trees were constructed based on either *rDNA ITS*, *EF-1α* or *RPB2* using MEGA 7.0.26. Clade support was inferred from 1000 bootstrap replicates and alignment gaps were excluded. The tree length (TL), consistency index (CI), retention index (RI) and rescaled consistency index (RCI) were also recorded. The constructed trees were edited using the online iTOL (https://itol.embl.de/). All amplified sequences were submitted directly to NCBI and given the accession numbers. 

### 4.4. Pathogenicity Assay of Fusarium Species on Soybean Seeds

Koch’s postulates were fulfilled to test the pathogenicity of identified *Fusarium* species using spore suspension inoculation as described by Naeem et al. [4]. The pathogenicity of three representative isolates of each *Fusarium* species were performed on healthy seeds of soybean cultivar Nandou12. Spore suspension was prepared through transferring 6−8 mycelium plugs into 30 mL of Carboxymethyl cellulose medium (CMC, 7.5 g∙L^−1^ carboxymethyl cellulose sodium, 0.5 g∙L^−1^ yeast extract, 2.5 g∙L^−1^ K_2_HPO_4_, and 0.25 g∙L^−1^ MgSO_4_) and incubated at an orbital rotator at 150 r∙min^−1^, 25 °C for 4 days to obtain a concentration of 1 × 10^5^ spores per mL. Ten soybean seeds were thoroughly surface-sterilized with 5% sodium hypochlorite, soaked in spore suspension for 5 min, and then incubated on PDA plates at 25 ± 2 °C in the darkness. Seeds were soaked in CMC medium as control. Three plates were prepared for each representative isolate, and three independent experiments were conducted. After seven-day inoculation, disease symptoms were observed and the disease severity grade was evaluated referred to the methods by Naeem et al. [4] with some modifications as follows: 0 = healthy seed germination without discoloration inside the seeds; 1 = delayed germination with negligible or no discoloration inside the seeds; 2 = low germination with slightly water-soaked and yellow symptoms inside the seeds; 3 = no germination with partially water-soaked, yellow or brown, softened decay inside the seeds; and 4 = no germination, brown and severe seed decay. The diseases index (DI) was calculated according to the formula below. In addition, seed fresh weight and germination rate were documented.

(1) $$DI=\frac{\sum \left(severitygrade\times seedspergrade\right)}{totalseeds\times thehighestseveritygrade}\times 100$$

### 4.5. Statistical Analysis

All data were recorded and processed using Microsoft office Excel. Mean values of disease index (DI), germination percentage and fresh weight of soybean seeds upon *Fusarium* isolates inoculation were obtained from 30 seeds of each isolate in three independent replicates. The data correlation was conducted by generalized linear model (GLM) with quasipoisson distribution for residuals, and statistical analysis was performed by Duncan’s test using SPSS Statistics 21 with significance difference at the level of P = 0.05. The isolation frequency for each *Fusarium* species in total *Fusarium* isolates and soybean varieties percentage colonized by *Fusarium* species were calculated based on total soybean varieties. Fisher’s exact test was used to analyze the difference of isolation frequency among *Fusarium* species and the difference of colonized varieties percentage among soybean varieties, respectively.

## 5. Conclusions

Seedborne diseases have emerged as one of the important yield-limiting factors for soybean production around the world. This study demonstrated that soybean seeds are commonly colonized by different fungal genera, especially *Fusarium* spp. Based on sequence analysis of multiple genes, five species in the *Fusarium* genus were identified from naturally infected soybean seeds in Sichuan Province of Southwest China, and there were significant difference in the isolation frequency, pathogenicity and cultivar-specificity of these *Fusarium* species. In addition, an infection relationship of *Fusarium* species between pods and seeds was preliminarily presented, but the virulence mechanism of these seedborne *Fusarium* species regarding to virulence factors, host range and pathogenicity needs to be elucidated in future work. To achieve this goal, the isolated fungi should be phenotypically characterized by analyzing the production of different virulence determinants such as toxin synthesis and biodegradation enzymes. Thus, in this study, the collection of soybean seedborne *Fusarium* species may contribute to advances on the effective disease management, and it will be valuable for the breeding of resistant materials against the dominant seedborne pathogens. 

## Supplementary Materials

The following are available online at https://www.mdpi.com/2076-0817/9/7/531/s1, Table S1: Information of the fungi isolates recovered from soybean seeds, identified species and GenBank accession numbers of *rDNA ITS*, *EF-1α* and *RPB2.* Figure S1: Symptoms infected by the other representative isolates of *Fusarium* species associated with soybean seed decay.
