# Supplementary material for: Diversity of the Seedborne Fungi and Pathogenicity of Fusarium Species Associated with Intercropped Soybean
Source: Pathogens. 2020 Jul 1;9(7):531. doi: 10.3390/pathogens9070531 (PMC7400112; doi:10.3390/pathogens9070531)
Supplement: Supplementary file 1 [file pathogens-09-00531-s001.pdf]

Supplementary material

Diversity of the seedborne fungi and pathogenicity of *Fusarium* species associated with intercropped soybean

Xiaoli Chang [1,2,†], Hongju Li [1,†], Muhammd Naeem [1,†], Xiaoling Wu [1], Taiwen Yong [1], Chun Song [1], Taiguo Liu[2,3], Wanquan Chen [2,3,*] and Wenyu Yang [1,*]

[1] College of Agronomy & Sichuan Engineering Research Center for Crop Strip Intercropping system, Sichuan Agricultural University, Chengdu 611130, China; xl_chang14042@sicau.edu.cn (X.C.); lihongju@stu.sicau.edu.cn (H.L.); muhammdnaeem201@gmail.com (M.N.); wulx@sicau.edu.cn (X.W.); yongtaiwen@sicau.edu.cn (T.Y.); songchun@sicau.edu.cn (C.S.); mssiyangwy@sicau.edu.cn (W.Y.)

[2] State Key Laboratory for Biology of Plant Diseases and Insect Pests, Institute of Plant Protection, Chinese Academy of Agricultural Sciences, Beijing 100193, China; xl_chang14042@sicau.edu.cn (X.C.); liutaiguo@caas.cn (T.L.); wqchen@ippcaas.cn (W.C.)

[3] National Agricultural Experimental Station for Plant Protection, Ministry of Agriculture and Rural Affairs, Gansu 741200, China; liutaiguo@caas.cn (T.L.); wqchen@ippcaas.cn (W.C.)

[†] These authors contributed equally to this work

* Correspondence: mssiyangwy@sicau.edu.cn (W.Y.), Tel: +86-28-86280870 (W.Y.), Fax: +86-10-86280872 (W.Y.); wqchen@ippcaas.cn (W.C.), Tel.: +86-10-62815618 (W.C.), Fax: +86-10-62895365 (W.C.)

Received: date; Accepted: date; Published: date

Table S1 Information of the fungi isolates recovered from soybean seeds, identified species and GenBank accession numbers of *rDNA ITS*, *EF-1α* and *RPB2*.

Isolates	Soybean varieties	Sampling locations	GenBank accession numbers			Fungal genera or species
			rDNA ITS	*EF1-α*	*RPB2*	
LS01	Gongqiu 4	Zigong	MN871538	MN901558	MN880735	*F. fujikuroi*
LS02	Gongxia 8173-ZLJ	Zigong	MN871539	MN901538	MN880736	*F. fujikuroi*
LS07	Gongxia 925	Zigong	MN871576	MN901527	MN880774	*F. fujikuroi*
LS09	Gongqiu 5-YT	Zigong	MN955506	MN901529	MN880737	*F. fujikuroi*
LS13	Gongqiu 5-YT	Zigong	MN955507	MN901535	MN880775	*F. fujikuroi*
LS16	Tongdou 11	Nanchong	MN871577	MN901537	MN880776	*F. fujikuroi*
LS18	Gongxia 9	Zigong	MN871542	MN901533	MN880738	*F. fujikuroi*
LS22	Gongqiu 5-YT	Zigong	MN871546	MN901546	MN880739	*F. fujikuroi*
LS23	Gongqiu 5-YT	Zigong	MN955508	MN901543	MN880772	*F. fujikuroi*

LS27	Gongxia 925	Zigong	MN955509	MN901540	MN880740	*F. fujikuroi*
LS30	Gongqiu 4	Zigong	MN871550	MN901561	MN880762	*F. fujikuroi*
LS31	Gongqiu 4	Zigong	MN871551	MN901542	MN880741	*F. fujikuroi*
LS35	Gongqiu 8	Zigong	MN871552	MN901560	MN880742	*F. fujikuroi*
LS36	Gongqiu 8	Zigong	MN955510	MN901544	MN880746	*F. fujikuroi*
LS37	Gongqiu 5-YT	Zigong	MN955511	MN901553	MN880747	*F. fujikuroi*
LS44	Gongxia 925	Zigong	MN955512	MN901525	MN880748	*F. fujikuroi*
LS45	Gongqiu 8	Zigong	MN871554	MN901545	MN880743	*F. fujikuroi*
LS46	Gongqiu 8	Zigong	MN955514	MN901541	MN880744	*F. fujikuroi*
LS47	Gongxia 925	Zigong	MN955513	MN901550	MN880749	*F. fujikuroi*
LS54	Gongxia8173-ZLJ	Zigong	MN955515	MN901530	MN880750	*F. fujikuroi*
LS58	Gongxia8173-ZLJ	Zigong	MN955516	MN901554	MN880751	*F. fujikuroi*
LS67	Gongqiu 4	Zigong	MN955517	MN901552	MN880752	*F. fujikuroi*
LS77	Tongdou 11	Nanchong	MN871583	MN901551	MN880753	*F. fujikuroi*
LS78	Tongdou 11	Nanchong	MN871584	MN901528	MN880754	*F. fujikuroi*
LS84	Gongxia369-1	Zigong	MN955518	MN901532	MN880773	*F. fujikuroi*
LS88	Gongxia369-1	Zigong	MN955519	MN901534	MN880755	*F. fujikuroi*
LS89	Gongxia369-1	Zigong	MN955520	MN901539	MN880756	*F. fujikuroi*
LS94	Gongqiu5-YT	Zigong	MN955521	MN901526	MN880771	*F. fujikuroi*
LS95	Gongqiu 5-BJ	Zigong	MN955522	MN901547	MN880757	*F. fujikuroi*
LS99	Gongxia 925	Zigong	MN955523	MN901524	MN880761	*F. fujikuroi*
LS100	Gongxia 925	Zigong	MN871588	MN901548	MN880758	*F. fujikuroi*
LS101	Gongxia 925	Zigong	MN871559	MN901531	MN880745	*F. fujikuroi*
LS104	Gongxia8173-GJ	Zigong	MN955524	MN901536	MN880759	*F. fujikuroi*
LS142	Gongqiu 5-YT	Zigong	MN871563	MN901555	MN880770	*F. fujikuroi*
LS148	Gongqiu 8	Zigong	MN955525	MN901563	MN880763	*F. fujikuroi*
LS159	Gongxia8173-GJ	Zigong	MN871566	MN901564	MN880764	*F. fujikuroi*
LS162	Gongxia8173-GJ	Zigong	MN871567	MN901556	MN880765	*F. fujikuroi*
LS166	Tongdou 11	Nanchong	MN955526	MN901549	MN880760	*F. fujikuroi*
LS167	Tongdou 11	Nanchong	MN955527	MN901562	MN880766	*F. fujikuroi*
LS177	Gongqiu 4	Zigong	MN871569	MN901557	MN880767	*F. fujikuroi*

LS199	Gongqiu 4	Zigong	MN955528	MN901559	MN880768	*F. fujikuroi*
LS200	Gongqiu 4	Zigong	MN871575	MN901565	MN880769	*F. fujikuroi*
LS08	Gongxia 925	Zigong	MN955529	MN901580	MN943312	*F. incarnatum*
LS19	Gongqiu 5-YT	Zigong	MN871543	MN901601	MN943321	*F. incarnatum*
LS24	Gongqiu 5-YT	Zigong	MN955530	MN901590	MN943328	*F. incarnatum*
LS25	Gongxia 369-1	Zigong	MN871547	MN901596	MN943309	*F. incarnatum*
LS26	Gongxia 369-1	Zigong	MN871548	MN901581	MN943310	*F. incarnatum*
LS28	Gongxia 925	Zigong	MN871549	MN901579	MN943311	*F. incarnatum*
LS29	Gongxia 925	Zigong	MN955531	MN901582	MN943322	*F. incarnatum*
LS32	Gongqiu 8	Zigong	MN955532	MN901588	MN943316	*F. incarnatum*
LS34	Gongqiu 8	Zigong	MN955533	MN901598	MN943313	*F. incarnatum*
LS40	Gongxia 8173-ZLJ	Zigong	MN871553	MN901583	MN943314	*F. incarnatum*
LS60	Gongqiu 5-YT	Zigong	MN871555	MN901595	MN943327	*F. incarnatum*
LS69	Gongqiu 8	Zigong	MN871578	MN901586	MN943315	*F. incarnatum*
LS72	Nandou 12	Chongzhou	MN871579	MN901587	MN943317	*F. incarnatum*
LS73	Tongdou 11	Nanchong	MN871580	MN901584	MN943318	*F. incarnatum*
LS760	Gongqiu 5-BJ	Zigong	MN871556	MN901597	MN943331	*F. incarnatum*
LS820	Gongxia 369-1	Zigong	MN871557	MN901592	MN943330	*F. incarnatum*
LS87	Gongxia 369-1	Zigong	MN871587	MN901591	MN943319	*F. incarnatum*
LS106	Gongqiu 4	Zigong	MN871560	MN901585	MN943320	*F. incarnatum*
LS139	Gongqiu 5-YT	Zigong	MN871562	MN901589	MN943326	*F. incarnatum*
LS153	Gongxia 8173-ZLJ	Zigong	MN871564	MN901594	MN943323	*F. incarnatum*
LS154	Gongxia 8173-ZLJ	Zigong	MN955534	MN901593	MN943324	*F. incarnatum*
LS157	Gongxia 8173-ZLJ	Zigong	MN871565	MN901599	MN943325	*F. incarnatum*
LS184	Nanxia	Nanchong	MN871571	MN901578	MN943332	*F. incarnatum*
LS186	Nanxia	Nanchong	MN871572	MN901600	MN943329	*F. incarnatum*
LS03	Tongdou 11	Nanchong	MN871540	MN901574	MN880782	*F. proliferatum*
LS20	Gongqiu 5-YT	Zigong	MN871544	MN901571	MN880783	*F. proliferatum*
LS91	Gongqiu 5-YT	Zigong	MN871558	MN901570	MN880784	*F. proliferatum*
LS137	Gongqiu 5-YT	Zigong	MN871561	MN901576	MN880781	*F. proliferatum*
LS181	Gongqiu 5-BJ	Zigong	MN871570	MN901577	MN880777	*F. proliferatum*

LS192	Gongqiu 5-YT	Zigong	MN955535	MN901573	MN880778	*F. proliferatum*
LS193	Gongqiu 5-YT	Zigong	MN871573	MN901572	MN880779	*F. proliferatum*
LS196	Gongqiu 5-YT	Zigong	MN955539	MN901575	MN880780	*F. proliferatum*
LS197	Gongqiu 5-YT	Zigong	MN871574	MN901569	MN880785	*F. proliferatum*
LS71	Nandou 12	Chongzhou	MN871581	MN901602	MN883833	*F. asiaticum*
LS76	Nandou 12	Chongzhou	MN871582	MN901603	MN883834	*F. asiaticum*
LS82	Nandou 12	Chongzhou	MN871585	MN901604	MN883835	*F. asiaticum*
LS86	Nandou 12	Chongzhou	MN871586	MN901605	MN883836	*F. asiaticum*
LS12	Gongxia 8173-GJ	Zigong	MN871541	MN901567	MN883830	*F. verticillioides*
LS21	Gongqiu 5-YT	Zigong	MN871545	MN901568	MN883832	*F. verticillioides*
LS172	Gongxia 369-1	Zigong	MN871568	MN901566	MN883831	*F. verticillioides*
1	Tongdou 11	Zigong	MN883902	–	–	*Colletotrichum* sp.
7	Gongxia 925	Zigong	MN883903	–	–	*Colletotrichum* sp.
11	Gongxia 8173-GJ	Zigong	MN883904	–	–	*Colletotrichum* sp.
42	Gongxia 8173-GJ	Zigong	MN883906	–	–	*Colletotrichum* sp.
47	Gongqiu 8	Zigong	MN883907	–	–	*Colletotrichum* sp.
55	Gongxia 8173-ZLJ	Zigong	MN883908	–	–	*Colletotrichum* sp.
83	Gongxia 369-1	Zigong	MN883909	–	–	*Colletotrichum* sp.
169	Gongxia 369-1	Zigong	MN883910	–	–	*Colletotrichum* sp.
171	Gongxia 369-1	Zigong	MN883911	–	–	*Colletotrichum* sp.
180	Gongqiu 4	Zigong	MN883912	–	–	*Colletotrichum* sp.
14	Tongdou 11	Zigong	MN883913	–	–	*Colletotrichum* sp.
15	Tongdou 11	Zigong	MN883914	–	–	*Colletotrichum* sp.
38	Gongqiu 5-BJ	Zigong	MN871589	–	–	*Colletotrichum* sp.
39	Gongxia 8173-ZLJ	Zigong	MN871590			*Colletotrichum* sp.
56	Gongxia 8173-ZLJ	Zigong	MN871591	–	–	*Colletotrichum* sp.
57	Gongxia 8173-ZLJ	Zigong	MN871592	–	–	*Colletotrichum* sp.
61	Gongqiu 5-YT	Zigong	MN871593	–	–	*Colletotrichum* sp.
80	Gongxia 369-1	Zigong	MN871594	–	–	*Colletotrichum* sp.
81	Gongxia 369-1	Zigong	MN871595	–	–	*Colletotrichum* sp.
103	Gongxia 925	Zigong	MN871596	–	–	*Colletotrichum* sp.

109	Gongqiu 4	Zigong	MN871597	–	–	*Colletotrichum* sp.
110	Gongqiu 4	Zigong	MN871598	–	–	*Colletotrichum* sp.
124	Gongqiu 5-BJ	Zigong	MN871599	–	–	*Colletotrichum* sp.
141	Gongqiu 5-YT	Zigong	MN871600	–	–	*Colletotrichum* sp.
175	Gongqiu 4	Zigong	MN871601	–	–	*Colletotrichum* sp.
145	Gongqiu 8	Zigong	MN871602	–	–	*Colletotrichum* sp.
155	Gongxia 8173-ZLJ	Zigong	MN871603	–	–	*Colletotrichum* sp.
168	Gongxia 369-1	Zigong	MN871604	–	–	*Colletotrichum* sp.
170	Gongxia 369-1	Zigong	MN871605	–	–	*Colletotrichum* sp.
176	Gongqiu 4	Zigong	MN871606	–	–	*Colletotrichum* sp.
179	Gongqiu 4	Zigong	MN871607	–	–	*Colletotrichum* sp.
183	Nanxia	Nanchong	MN871608	–	–	*Colletotrichum* sp.
189	Gongxia 8173-ZLJ	Zigong	MN871609	–	–	*Colletotrichum* sp.
43	Gongqiu 8	Zigong	MN871610	–	–	*Alternaria* sp.
74	Gongqiu 5-BJ	Zigong	MN871611	–	–	*Alternaria* sp.
104	Gongxia 925	Zigong	MN871612	–	–	*Alternaria* sp.
105	Gongxia 925	Zigong	MN871613	–	–	*Alternaria* sp.
114	Gongqiu 8	Zigong	MN871614	–	–	*Alternaria* sp.
150	Gongqiu 8	Zigong	MN871615	–	–	*Alternaria* sp.
185	Nanxia	Nanchong	MN871616	–	–	*Alternaria* sp.
29	Gongqiu 4	Zigong	MN871617	–	–	*Corynespora* sp.
86	Gongxia 369-1	Zigong	MN871618	–	–	*Corynespora* sp.
131	Gongxia 925	Zigong	MN871619	–	–	*Corynespora* sp.
96	Tongdou 11	Zigong	MN871620	–	–	*Corynespora* sp.
138	Gongqiu 5-YT	Zigong	MN871621	–	–	*Corynespora* sp.
147	Gongqiu 8	Zigong	MN871622	–	–	*Corynespora* sp.
44	Gongxia 8173-ZLJ	Zigong	MN871623	–	–	*Diaporthe*/*Phomopsis* sp.
62	Nandou 12	Chongzhou	MN871639	–	–	*Diaporthe*/*Phomopsis* sp.
107	Gongqiu 4	Zigong	MN871624	–	–	*Diaporthe*/*Phomopsis* sp.

111	Gongqiu 4	Zigong	MN871625	–	–	*Diaporthe/Phomopsis* sp.
117	Tongdou 11	Zigong	MN871626	–	–	*Diaporthe/Phomopsis* sp.
151	Nandou 12	Chongzhou	MN871627	–	–	*Diaporthe/Phomopsis* sp.
163	Tongdou 11	Zigong	MN871634	–	–	*Diaporthe/Phomopsis* sp.
37	Gongqiu 8	Zigong	MN871628	–	–	*Stagonosporopsis* sp.
113	Gongqiu 8	Zigong	MN883901	–	–	*Stagonosporopsis* sp.
178	Gongqiu 4	Zigong	MN871629	–	–	*Stagonosporopsis* sp.
17	Gongxia 9	Zigong	MN871630	–	–	*Chaetomium* sp.
36	Gongqiu 8	Zigong	MN871631	–	–	*Chaetomium* sp.
72	Gongxia 369-1	Zigong	MN871632	–	–	*Chaetomium* sp.
128	Gongxia 8173-GJ	Zigong	MN871633	–	–	*Didymella* sp.
6	Gongqiu 5-BJ	Zigong	MN871635	–	–	*Trichoderma* sp.
182	Gongxia 9	Zigong	MN871636	–	–	*Podospora* sp.
48	Gongxia 9	Zigong	MN871637	–	–	*Podospora* sp.
10	Gongxia 8173-GJ	Zigong	MN871638	–	–	*Macrophomina* sp.
41	Gongqiu 5-YT	Zigong	MN871640	–	–	*Thielavia* sp.
164	Tongdou 11	Zigong	MN871641	–	–	*Botryosphaeria* sp.

Notes: *rDNA ITS, ribosomal internal transcribed spacer region*; *EF-1α, translation elongation factor 1α* gene; *RPB2, RNA polymerase II second largest subunit* gene. " – " means gene sequences of *EF-1 α* or *RPB2* were not amplified and analyzed for these isolates.

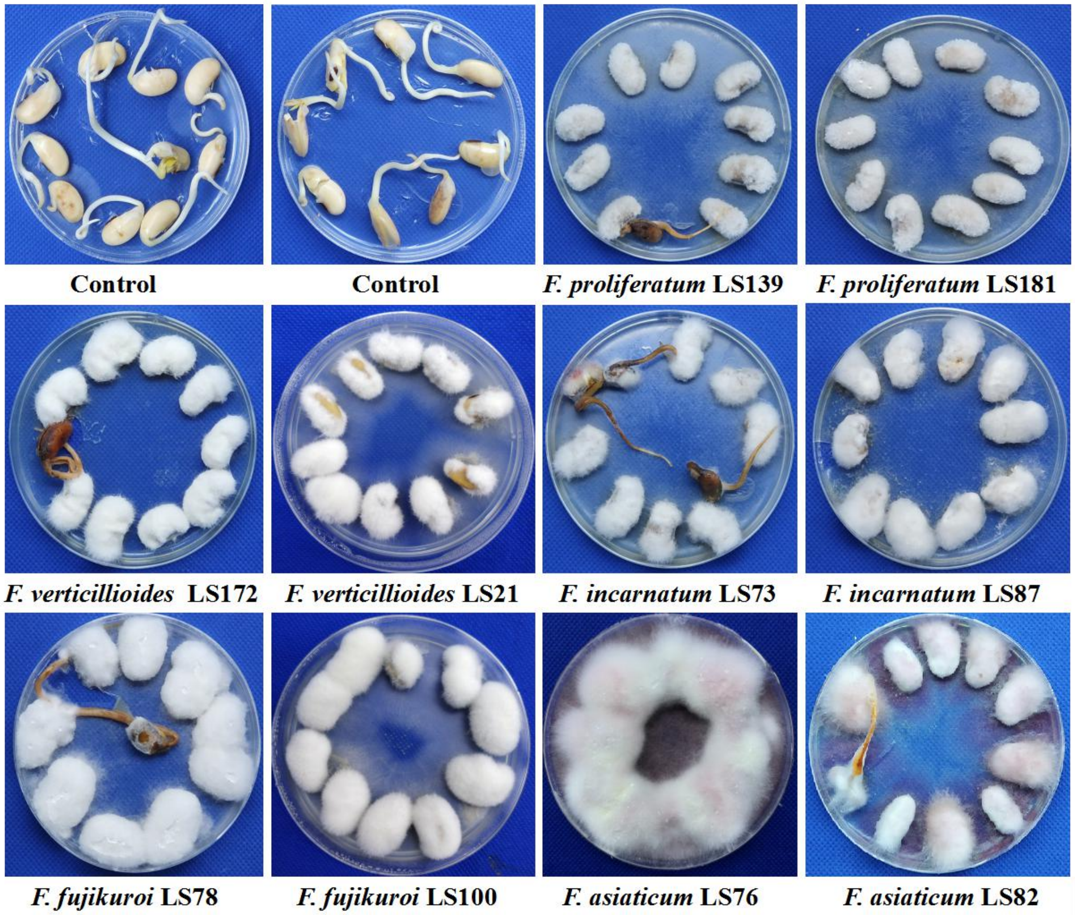

Figure S1. Symptoms infected by the other representative isolates of *Fusarium* species associated with soybean seed decay. The representative isolates include *F. proliferatum* LS137 and LS181; *F. fujikuroi* LS78 and LS100; *F. verticillioides* LS172 and LS21; *F. asiaticum* LS76 and LS82; and *F. incarnatum* LS73 and LS87; Control means the seeds inoculated with PDA medium instead of *Fusarium* isolates. After seven-day culture on PDA plates, mycelium growth of *Fusarium* species on the seed surface and the infection symptoms inside the seeds were observed.
